# A validation study of UCSD-Mayo risk score in predicting hospital-acquired acute kidney injury in COVID-19 patients

**DOI:** 10.1080/0886022X.2021.1948429

**Published:** 2021-07-07

**Authors:** Zhengying Fang, Chenni Gao, Yikai Cai, Lin Lu, Haijin Yu, Hafiz Muhammad Jafar Hussain, Zijin Chen, Chuanlei Li, Wenjie Wei, Yuhan Huang, Xiang Li, Shuwen Yu, Yinhong Ji, Qinjie Weng, Yan Ouyang, Xiaofan Hu, Jun Tong, Jian Liu, Mingyu Liu, Xiaoman Xu, Yixin Zha, Zhiyin Ye, Tingting Jiang, Jieshuang Jia, Jialin Liu, Yufang Bi, Nan Chen, Weiguo Hu, Huiming Wang, Jun Liu, Jingyuan Xie

**Affiliations:** a Department of Nephrology, Institute of Nephrology, Ruijin Hospital, Shanghai Jiao Tong University, School of Medicine, Shanghai, PR China; b Department of Nephrology, North Huashan Hospital, Fudan University, Shanghai, PR China; c Department of Nephrology, Shanghai General Hospital, Shanghai Jiao Tong University, Shanghai, PR China; d Wuhan Ninth Hospital, Wuhan, PR China; e Renal Department, Wuhan Ninth Hospital, Wuhan, PR China; f Clinical Research Center, Shanghai General Hospital, Shanghai Jiao Tong University, Shanghai, PR China; g Department of Critical Care Medicine, Ruijin Hospital, Shanghai Jiao Tong University, School of medicine, Shanghai, PR China; h Department of Endocrinology and Metabolism disease, Ruijin Hospital, Shanghai Jiao Tong University, School of Medicine, Shanghai, PR China; i Department of Surgery, Ruijin Hospital, Shanghai Jiao Tong University School of Medicine, Shanghai, PR China; j Renal Department of Renmin Hospital, Renmin Hospital of Wuhan University, Wuhan, PR China

**Keywords:** COVID-19, proximal tubule, acute kidney injury, risk factors

## Abstract

**Introduction:**

Acute kidney injury (AKI) in coronavirus disease 2019 (COVID-19) patients is associated with poor prognosis. Early prediction and intervention of AKI are vital for improving clinical outcome of COVID-19 patients. As lack of tools for early AKI detection in COVID-19 patients, this study aimed to validate the USCD-Mayo risk score in predicting hospital-acquired AKI in an extended multi-center COVID-19 cohort.

**Methods:**

Five hundred seventy-two COVID-19 patients from Wuhan Tongji Hospital Guanggu Branch, Wuhan Leishenshan Hospital, and Wuhan No. Ninth Hospital was enrolled for this study. Patients who developed AKI or reached an outcome of recovery or death during the study period were included. Predictors were evaluated according to data extracted from medical records.

**Results:**

Of all patients, a total of 44 (8%) developed AKI. The UCSD-Mayo risk score achieved excellent discrimination in predicting AKI with the C-statistic of 0.88 (95%CI: 0.84–0.91). Next, we determined the UCSD-Mayo risk score had good overall performance (Nagelkerke *R*^2^ = 0.32) and calibration in our cohort. Further analysis showed that the UCSD-Mayo risk score performed well in subgroups defined by gender, age, and several chronic comorbidities. However, the discrimination of the UCSD-Mayo risk score in ICU patients and patients with mechanical ventilation was not good which might be resulted from different risk factors of these patients.

**Conclusions:**

We validated the performance of UCSD-Mayo risk score in predicting hospital-acquired AKI in COVID-19 patients was excellent except for patients from ICU or patients with mechanical ventilation.

## Introduction

Coronavirus disease 2019 (COVID-19) is a systemic disease caused by severe acute respiratory syndrome coronavirus 2 (SARS-CoV-2). Acute kidney injury (AKI) is common among COVID-19 patients, but studies reported highly varying rates of AKI (0.5–36.6%) previously [1–5]. An earlier study based on Chinese patients from Wuhan reported 5.1% of AKI development in hospitalized COVID-19 patients [2]. Another study based on patients from USA showed AKI occurrence in 36.6% hospitalized COVID-19 patients [3]. The reason(s) for this large difference in AKI incidence among COVID-19 patients is not clear yet, but it might be because of the differences in the definition(s) of AKI, the proportion of severely ill patients, and ethnicity in these studies. In both studies, patients who developed AKI were more likely to experience poor prognosis including a higher risk of death [2,3]. Therefore, early prediction, detection, and intervention of AKI in COVID-19 patients are important to improve their outcome.

The mechanism of COVID-19 related AKI is not clear yet. Direct injury by SARS-CoV-2 as well as indirect mechanisms including systemic inflammation, hemodynamics change, and mechanical ventilation may contribute to AKI development [6]. Acute tubular necrosis is predominant in COVID-19 patients revealed by renal biopsy as well as postmodern studies [7–9]. Other pathologic changes of the kidney include collapsing and crescentic glomerulopathies, membranous nephropathy, and thrombotic microangiopathy [9,10]. The differences in pathological types suggest that the mechanism of kidney injury is complex. Furthermore, since the prevalence of AKI is significantly higher within COVID-19 patients from intensive care unit (ICU), indicating different mechanisms of AKI between ICU and non-ICU patients [11].

Previous studies have identified several risk factors of AKI among COVID-19 patients, including older age, male gender as well as basic comorbidities such as hypertension and diabetes mellitus [12–14]. Besides, mechanical ventilation is a strong predictor for AKI which has been proved in a large cohort study [3]. Laboratory test results can also predict the occurrence of AKI, such as elevated soluble urokinase receptor level, which may promote mitochondrial superoxide generation [15,16]. As each factor alone is insufficient to predict the overall risk of AKI development, combining many risk factors to establish a risk score can predict the occurrence of AKI more accurately. There are few studies on the AKI risk score of COVID-19 patients. One small sample-size study developed a prediction model for COVID-19 related AKI including gender, blood urea nitrogen (BUN), and cortex-to-aorta enhancement index resulted from contrast-enhanced CT scan [17]. However, considering the medical conditions and risks of contract agents, it is not possible to perform an enhanced CT scan for every COVID-19 patient.

The UCSD-Mayo risk score was established for predicting AKI in the ICU based on clinical parameters at the time of admission. A risk prediction score driven from this model includes laboratory tests and chronic comorbidities [18]. Predictors in UCSD-Mayo risk score are simple, objective, and feasible, which can be obtained easily on admission. The renal angina index is another AKI prediction tool requiring multiple measurements of serum creatinine [19,20]. Considering the UCSD-Mayo risk score can be used to predict the risk of AKI at the time of admission, this study aims to validate the performance of the UCSD-Mayo risk score by our extended cohort consisting of Chinese patients with COVID-19.

## Materials and methods

### Study design and cohort

We retrospectively reviewed the medical records of all COVID-19 patients admitted to three hospitals in China from 1 February 2020 to 20 March 2020. The three hospitals were Tongji Hospital Guanggu Branch, Leishenshan Hospital, and No. Ninth Hospital. The three hospitals were designated hospital in Wuhan responsible for the treatments of patients with COVID-19. All the COVID-19 patients recruited in this study were tested SARS-CoV-2 by throat-swab specimens from the upper respiratory tract. Real-time reverse transcription polymerase chain reaction (RT-PCR) assay was used to confirm the COVID-19 and exclude other viral infections [21]. Recruitment criteria included: (1) patients diagnosed as COVID-19 according to World Health Organization guidance; (2) age equal to or older than 14 years; (3) clinical and follow-up data were available; (4) at least two serum creatinine measurements during hospitalization were available; and (5) patients reached the primary endpoint or dead or recovered from COVID-19 infection defined by at least three times negative results of SARS-CoV-2 testing during the study period. Patients who did not develop the primary endpoint and did not reach an outcome of death or recovery were not included in the analysis.

### Data collection and outcome

Data extracted from the medical records included patient demographic information, comorbidities, examination results, treatments (including mechanical ventilation and renal replacement therapy), and outcomes. Medications before admission were reported by patients. Predictors were evaluated according to the data mentioned above and laboratory tests within 48 h of admission. The primary endpoint was the time of the development of AKI during hospitalization.

### Definition

AKI was defined as an increase in serum creatinine by 0.3 mg/dL within 48 h or a 50% increase in serum creatinine from baseline within 7 d according to the KDIGO criteria [22]. End-stage renal disease was defined as first dialysis or kidney transplantation.

The UCSD-Mayo risk score was calculated by the original equation: Probability of AKI = e*^a^*/(1 + e*^a^*), where *a* = (0.059 + [0.860 × chronic kidney disease] + [0.778 × chronic liver disease] + [0.720× chronic heart failure] + [0.563 × hypertension] + [0.490 × atherosclerotic coronary vascular disease] + [0.977× acidosis] + [0.929 × nephrotoxin exposure] + [0.743 × severe infection] + [0.447 × mechanical ventilation] + [0.390 × anemia] [18]. The definition of predictors are based on the original article and the details are as followed: chronic kidney disease, stage 3 and 4 were calculated using Chronic Kidney Disease Epidemiology Collaboration equation; chronic liver disease, history of viral hepatitis, auto-immune hepatitis, non-alcohol fatty liver disease, cirrhosis, or liver failure as listed in the electronic health record as problem list; chronic heart failure, history of chronic heart failure or abnormal findings based on physical examination and X-ray/chest computed tomography or left ventricular ejection fractions <50%; hypertension, history of hypertension, or patients receiving chronic hypertensive medications; atherosclerotic coronary vascular disease, antecedent of angina pectoris, coronary artery disease, myocardial infarction, or peripheral vascular disease; acidosis, blood pH value ≤7.30, or serum bicarbonate value <2 1mmol/L; nephrotoxin exposure, exposure to radio-contrast or ≥3 nephrotoxins (amphotericin B, aminoglycosides, chemotherapy, anti-retroviral drugs, nonsteroidal anti-inflammatory drugs excluding aspirin) at once or ≥3 d of aminoglycoside therapy within 7 d prior to admission; severe infection, with two of these findings: respiratory rate >20/min, pulse >90/min, temperature >38 °C or <36 °C, white blood cell count >12,000/mL or <4000/mL; mechanical ventilation, need of mechanical ventilator; anemia, hemoglobin value less than 90 g/L or hematocrit value less than 27%.

### Statistical analyses

Baseline clinical variables were assessed for normality. Normally distributed variables were summarized using mean and standard deviation. Non-normally distributed continuous variables were expressed as median (interquartile range). Categorical variables were expressed as absolute (*n*) and percentage. The primary outcome was time for AKI development. Patients were followed up until AKI development, death or recovery from COVID-19. Death or recovery without AKI development was a censoring event and AKI development was a competing event. The association of predictors of UCSD-Mayo risk score with the primary outcome was tested by cox regression analysis. The UCSD-Mayo risk score was ascertained using a receiver-operating characteristic (ROC) curve analysis and calibration curve analysis. Nagelkerke *R*^2^ was also calculated. Statistical analysis was performed using Package pROC version 1.16.2; Package survival version 3.2–7; Package survminer version 0.4.8; Package rms version 6.0–1 (R version 4.0.2, R Project for Statistical Computing, Vienna, Austria).

## Results

### Comparison of baseline characteristics and outcome between the validation cohort and original cohort

**Figure 1.**
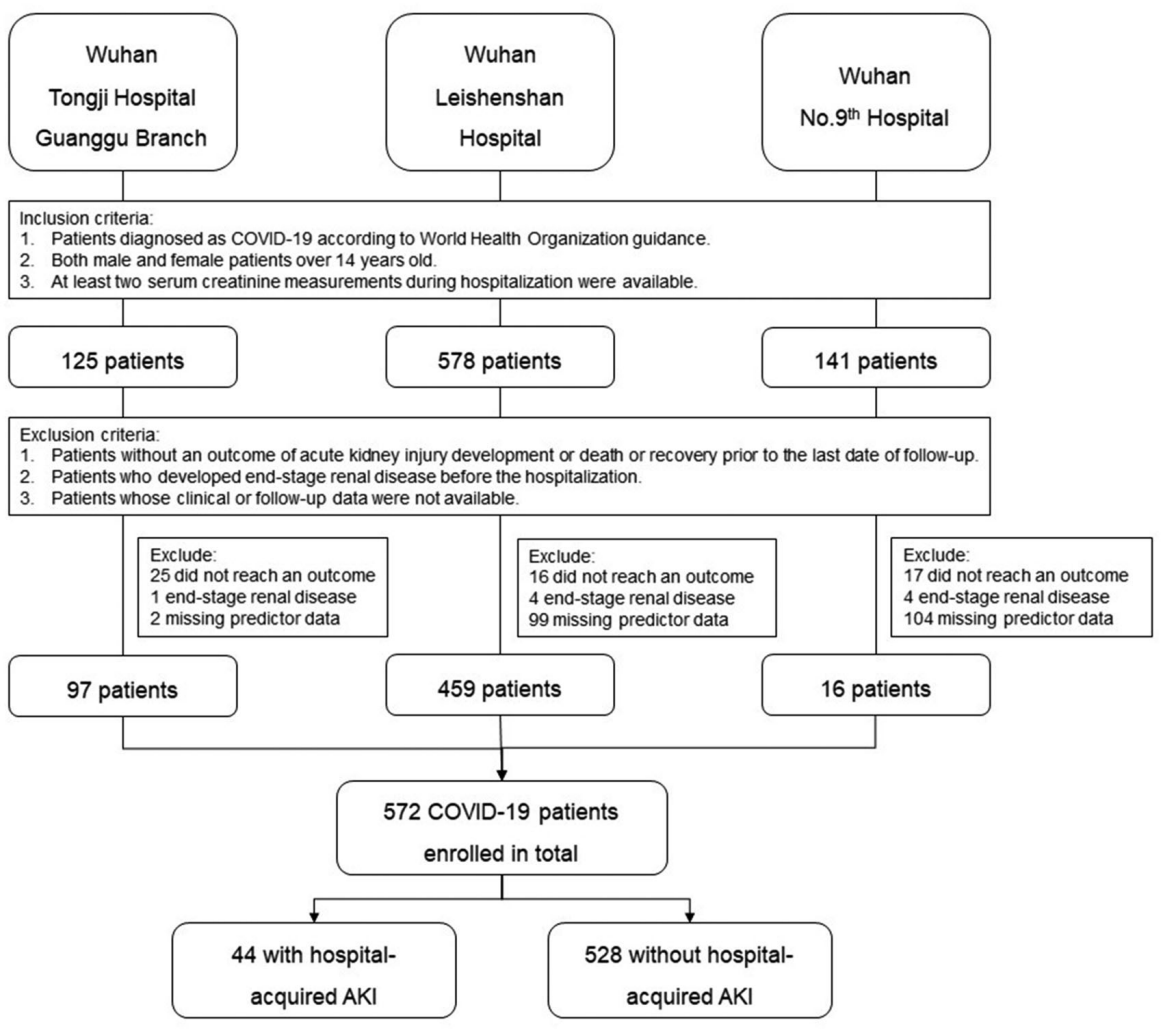
The flowchart of the enrolled patients.

The baseline demographic and outcome of COVID-19 cohort, as well as the comparison with an original cohort, are presented in Table 1. The COVID-19 validation cohort is composed of 572 patients who met the criteria for analysis (Figure 1). Patients with incomplete data on predictors of UCSD-Mayo risk score or clinical outcomes were excluded. Compared with patients in the UCSD development cohort, patients in the COVID-19 validation cohort had a significantly lower rate of severe infection, nephrotoxin exposure, and mechanical ventilation, reflecting worse overall conditions in the development cohort than in the COVID-19 validation cohort. Besides, the incidence of AKI in the COVID-19 cohort is 8%, which is lower than the UCSD cohort. However, the mortality rate of the COVID-19 cohort is higher than that of the UCSD cohort. The disparity may result from different study subjects, as the UCSD development cohort included patients from surgical intensive units and medical intensive units while the COVID-19 validation cohort enrolled hospitalized patients with confirmed COVID-19 diagnosis (Table 1).

**Table 1.** Demographic and outcome of patients from COVID-19 validation cohort and UCSD development cohort.

Variables	COVID-19 validation cohort	UCSD development cohort	*p* Value
	(*n* = 572)	(*n* = 573)	
Age (years)	61 ± 14	54 ± 18	NA
Ag*e* > 70, *n* (%)	144 (25)	120 (21)	.09
Male, *n* (%)	300 (52)	367 (64)	<.01
Hypertension, *n* (%)	236 (41)	207 (36)	.07
CLD, *n* (%)	68 (12)	61 (11)	.51
CHF, *n* (%)	63 (11)	77 (13)	.21
CKD, *n* (%)	28 (5)	43 (8)	.07
ASCVD, *n* (%)	57 (10)	NA	NA
Anemia, *n* (%)	24 (4)	NA	NA
Acidosis, *n* (%)	62 (11)	79 (14)	.13
Severe infection, *n* (%)	48 (8)	93 (16)	<.01
Nephrotoxin exposure, *n* (%)	8 (1)	114 (20)	<.01
Mechanical ventilation, *n* (%)	14 (2)	238 (42)	<.01
SCr at admission, mg/dL	0.8 (0.6-0.9)	0.9 (0.7–1.1)	NA
Incidence of AKI, *n* (%)	44 (8)	127 (22)	<.01
Need for RRT, *n* (%)	18 (3)	30 (5)	.08
Mortality, *n* (%)	66 (12)	41 (7)	.01

COVID-19: coronavirus disease 2019; SCr: serum creatinine; CLD: chronic liver disease; CHF: congestive heart failure; CKD: chronic kidney disease; ASCVD: atherosclerotic coronary vascular disease; RRT: renal replacement therapy; AKI: acute kidney injury: an increase in serum creatinine by 0.3 mg/dL within 48 h or a 50% increase in serum creatinine from baseline within 7 d.

### Survival analysis of the predictors and risk score by the validation cohort

**Figure 2.**
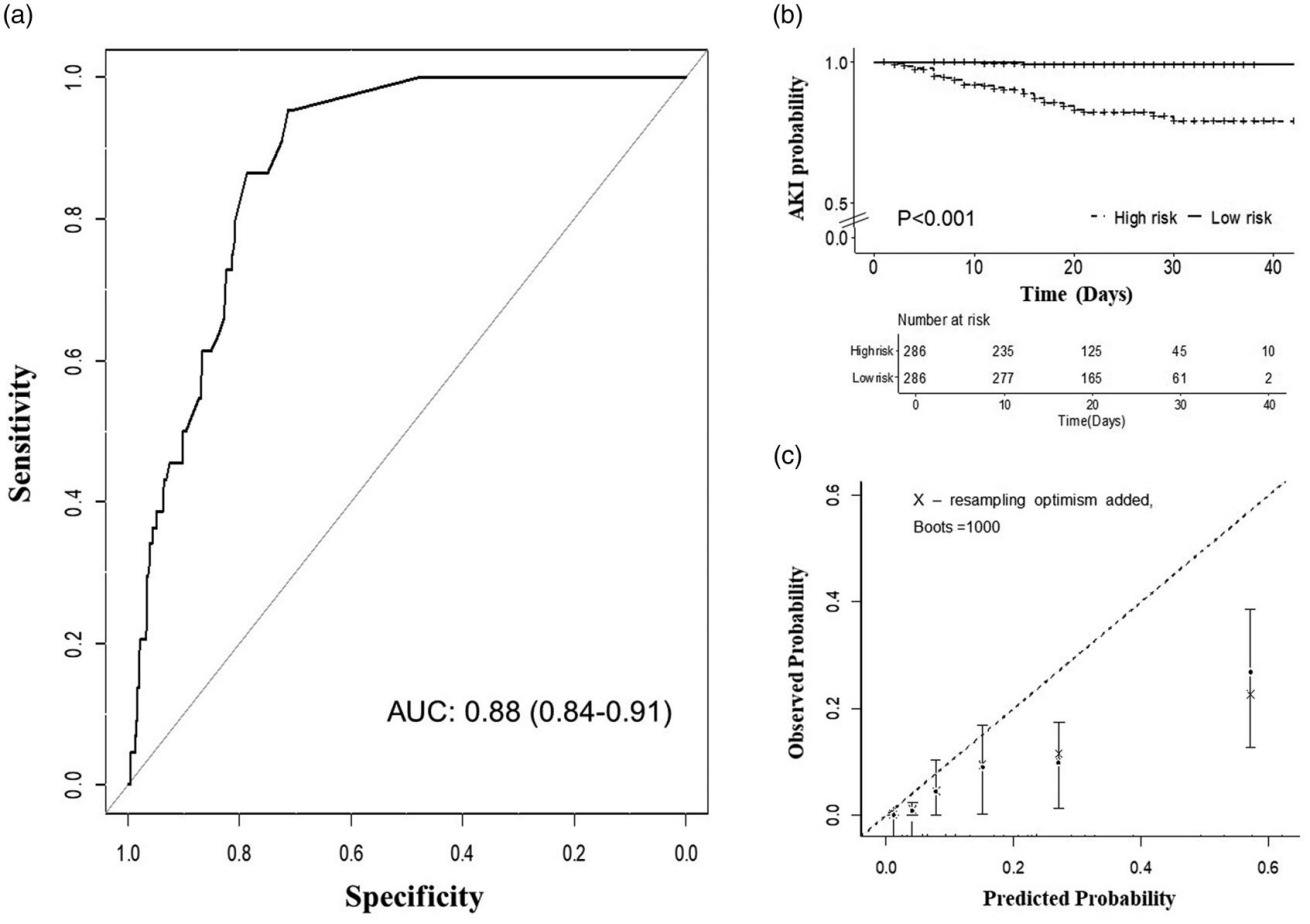
Validation of UCSD-Mayo Model in the COVID-19 cohort. (a) Area under curve of UCSD-Mayo risk score for prediction of AKI in COVID-19 validation cohort. (b) Kaplan–Meier curve with AKI-free survival of patients (*n* = 572) according to their risk group. (c) Calibration curve of UCSD-Mayo risk score for prediction of acute kidney injury in COVID-19 validation cohort.

Predictors in the UCSD-Mayo risk score were validated by univariate cox regression analysis in the COVID-19 cohort (Table 2). All the predictors involved in the UCSD-Mayo risk score are associated with a higher risk of AKI development, except for chronic kidney disease and nephrotoxin exposure which may due to the relatively low frequency. According to the probability of AKI calculated by the UCSD-Mayo risk score, we divided the COVID-19 patients into the high-risk group and the low-risk group. Cox regression analysis showed that patients in the high-risk group had a significantly increased risk of AKI development compared to the low-risk group (log-rank test, *p* < .001) (Figure 2(b)).

**Table 2.** Cox regression analysis of predictors from the UCSD-Mayo model (*n* = 572 patients).

Predictors	AKI patients	AKI-free patients	HR [95%CI]
	(*n* = 44)	(*n* = 528)	
Hypertension, *n* (%)	28 (64)	208 (39)	2.73 (1.48–5.04)*
CLD, *n* (%)	15 (34)	53 (10)	4.74 (2.54–8.86)*
CHF, *n* (%)	25 (57)	38 (7)	14.8 (8.13–26.96)*
CKD, *n* (%)	2 (5)	26 (5)	1.06 (0.26–4.37)
ASCVD, *n* (%)	12 (27)	45 (9)	4.03 (2.07–7.82)*
Anemia, *n* (%)	6 (14)	18 (3)	4.08 (1.72–9.65)*
Acidosis, *n* (%)	13 (30)	49 (9)	4.09 (2.14–7.81)*
Severe infection, *n* (%)	14 (32)	34 (6)	6.74 (3.57–12.73)*
Nephrotoxin exposure, *n* (%)	2 (5)	6 (1)	3.02 (0.73–12.49)
Mechanical ventilation, *n* (%)	9 (20)	5 (10)	23.89 (11.31–50.45)*

*With a *p* value < .01.

CLD: chronic liver disease; CHF: congestive heart failure; CKD: chronic kidney disease; ASCVD: atherosclerotic coronary vascular disease; AKI: acute kidney injury: an increase in serum creatinine by 0.3 mg/dL within 48 h or a 50% increase in serum creatinine from baseline within 7 d.

### Measures of discrimination and model fit

The discrimination of the UCSD-Mayo risk score in the COVID-19 validation cohort is measured by ROC curve analysis and reached an area under the ROC curves (AUROC) of 0.88 (95% confidential interval [CI]: 0.84–0.91), suggesting excellent discrimination (Figure 2(a)). The UCSD-Mayo risk score showed overall good performance (Nagelkerke *R*^2^ = 0.32) and calibration, with a reasonable agreement between observed and predicted AKI development in the COVID-19 validation cohort (Hosmer–Lemeshow test: chi-square 14.59, *p* = .10) (Figure 2(c)).

### Subgroup analyses

**Figure 3.**
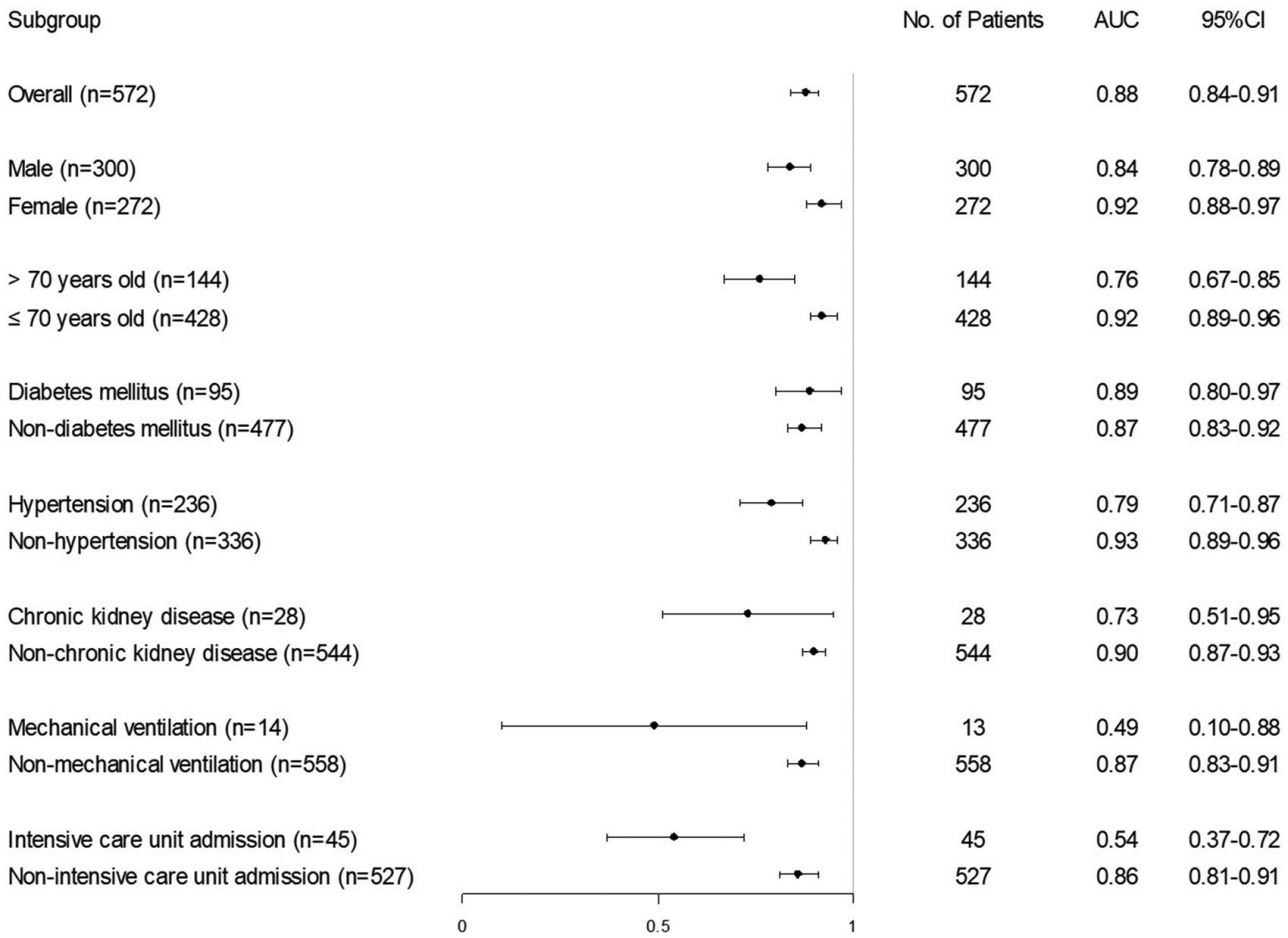
Subgroup analysis of UCSD-Mayo model in COVID-19 cohort.

Subgroup analysis was based on risk factors for COVID-19 related AKI. We found that the UCSD-Mayo risk score performed better in females, young (age ≤ 70 years old), non-hypertensive patients, and the patients without chronic kidney disease. In other subgroups defined by age, gender, and several clinical parameters, the performance of the UCSD-Mayo risk score is similar, except in ICU patients and mechanical ventilation patients (Figure 3). Further analysis revealed that great differences in clinical features between ICU and non-ICU admission patients may be an underlying cause. ICU admitted patients were older, suffering more chronic comorbidities including hypertension, chronic liver disease, and atherosclerotic coronary vascular disease as well as had a more severe infection on the time of admission. The rates of AKI development and mortality in ICU admitted patients were higher, 58% and 87%, respectively (Table 3).

**Table 3.** Clinical features of patients with ICU and non-ICU admission.

Variables	ICU admission	non-ICU admission	*p* Value
	(*n* = 45)	(*n* = 527)	
Age, years, mean (SD)	70 ± 11	61 ± 14	
Age >70, *n* (%)	23 (51)	121 (23)	<.01
Male, *n* (%)	27 (60)	273 (52)	.30
Hypertension, *n* (%)	31 (69)	205 (39)	<.01
CLD, *n* (%)	19 (42)	49 (9)	<.01
CHF, *n* (%)	27 (60)	36 (7)	<.01
CKD, *n* (%)	5 (11)	23 (4)	.10
ASCVD, n (%)	15 (33)	24 (5)	<.01
Anemia, *n* (%)	5 (11)	19 (4)	.04
Acidosis, *n* (%)	15 (33)	47 (9)	<.01
Severe infection, *n* (%)	13 (29)	35 (7)	<.01
Nephrotoxin exposure, *n* (%)	1 (0.02)	1 (0.002)	<.01
Mechanical ventilation, *n* (%)	14 (31)	0 (0)	<.01
SCr at admission, mg/dL	0.9 (0.6–1.2)	0.7 (0.6–0.9)	
Incidence of AKI, *n* (%)	26 (58)	18 (3)	<.01
AKI stage 1, *n* (%)	11 (24)	6 (1)	<.01
AKI stage 2, *n* (%)	7 (16)	6 (1)	<.01
AKI stage 3, *n* (%)	8 (18)	6 (1)	<.01
Need for RRT, *n* (%)	10 (22)	8 (2)	<.01
Mortality, *n* (%)	39 (87)	27 (5)	<.01

ICU: intensive care unit; CLD: chronic liver disease; CHF: congestive heart failure; CKD: chronic kidney disease; ASCVD: atherosclerosis coronary vascular disease; RRT: renal replacement therapy; AKI: acute kidney injury: an increase in serum creatinine by 0.3 mg/dL within 48 h or a 50% increase in serum creatinine from baseline within 7 d.

## Discussion

In-hospital AKI is common and associated with high mortality in COVID-19 patients [1–5]. Thus, early detection and prevention for AKI are vital to improve the clinical outcomes of COVID-19 patients. Currently, several AKI risk models have been developed and among them, the UCSD-Mayo risk score is a well-acknowledged model with ten predictors including acute and chronic risk factors which are simple and objective [18]. However, it is unknown whether we can use the UCSD-Mayo risk score for AKI prediction in COVID-19 patients. A higher rate of AKI has been reported in COVID-19 patients compared to patients with similar traditional risk factors. Besides, COVID-19 was found independently associated with a high rate of AKI after fully adjusted by traditional risk factors [23]. Based on these findings, validation studies of previous AKI risk scores are necessary before they can be used to predict AKI in COVID-19 patients.

In our study, the incidence of hospital-acquired AKI was 8%, being consistent with a meta-analysis including 5479 patients from 26 studies which found a pooled incidence of AKI of 8.4% in COVID-19 patients [24]. However, this rate is much lower than those previously reported from the USA [3,25]. The difference in the incidence of AKI is partly caused by disease severity and mechanical ventilation of the recruited patients, as mechanical ventilation has been proved to be strongly related to COVID-19 associated AKI [3]. Beside this, a higher rate of comorbidities including hypertension and diabetes mellitus might cause the difference.

In this study, we provide validation of the UCSD-Mayo risk score for hospital-acquired AKI by Chinese COVID-19 patients. Although patients with COVID-19 carry many risk factors for AKI including acute respiratory distress syndrome, mechanical ventilation, and systemic inflammation. Interestingly, still it is unclear whether patients with COVID-19 had unique risk factors beyond traditional AKI risk factors. A recent cohort study compared the AKI rates between patients with or without COVID-19 based on an extended cohort study including 22,122 hospitalized patients of whom 2600 tested positive for SARS-CoV-2. The authors found COVID-19 was independently associated with higher rates of AKI (HR = 1.4, 95%CI 1.29–1.53) after fully adjusted by traditional AKI risk factors suggesting the presence of a unique mechanism of COVID-19 which may include a direct effect of COVID-19 [23]. Based on these findings, a validation study of any previous AKI risk score is necessary before it can be used to predict AKI in COVID-19 patients. In our validation cohort, the predictors in the UCSD-Mayo risk score were associated with an increased risk of AKI. The UCSD-Mayo risk score showed an excellent performance in predicting AKI for hospitalized COVID-19 patients in terms of overall performance (Nagelkerke *R*^2^) and discrimination (C statistics). The AUROC of the USCD-Mayo risk score in the COVID-19 validation cohort reached 0.88 (95% confidential interval [CI]: 0.84–0.91), suggesting similar performance than that in the development data [18]. When we divided the patients into high and low-risk groups by the median of risk score, patients in the high-risk group had a significantly increased risk of AKI development compared to the low-risk group. Additionally, the risk prediction model showed good calibration, with a reasonable agreement between observed and predicted AKI outcome in the COVID-19 cohort. All these results determined the UCSD-Mayo risk score can be used to predict AKI in COVID-19 patients.

Moreover, subgroup analysis revealed that the UCSD-Mayo risk score has good performance in predicting COVID-19 associated AKI in different subgroups defined by gender, age, and common comorbidities. However, the risk score performed worse in the mechanical ventilation and ICU subgroups in predicting AKI in COVID-19 patients. We found that compared to non-ICU patients, ICU patients were older, had more chronic comorbidities, more severe infection, and higher mortality as well. The incidence of AKI in patients with ICU admission was much higher than with non-ICU admission (58% *vs.* 1%). Our findings indicate that the main cause, mechanism, and risk factors of COVID-19 associated AKI may be different among ICU or non-ICU patients. For ICU patients, mechanical ventilation, systemic inflammation, immune dysfunction, and vasoconstrictor drugs may be the main mechanism for AKI. On the other hand, these risk factors may not play a major role in non-ICU patients. Alternatively, direct viral effects on the kidney including tubular necrosis and coagulopathy might play a more important role in these patients. The USCD-Mayo risk score was established for predicting AKI in the ICU, however, it is not sensitive to predict the development of AKI in ICU patients in our study. There might have several underlying reasons. First, there was a difference between the basic diseases in the two cohorts. All patients enrolled in our study were COVID-19 patients, which are a specific group of patients and are not supposed to be included in the original develop cohort. Second, only 45 ICU patients were enrolled in our study, therefore, the validation in the ICU subgroup may be under power and remains to be further confirmed in a larger cohort with more ICU patients. Our findings indicate new risk factors might cooperate in the risk score for predicting AKI in ICU patients or patients with mechanical ventilation.

Our study has several strengths. First, as far as we know, this study is the first study on validation UCSD-Mayo risk score in Chinese patients with COVID-19, aiming at the early prediction of AKI within 48 h of admission. Besides, we enrolled an extended cohort from major COVID-19 designated hospitals in Wuhan. Lastly, the definition of AKI strictly follows the KDIGO guidelines and all patients have at least two measurements of renal function after admission. Our study also has several limitations. First, due to the retrospective design of our study, the frequency and number of assessments of renal function were different for each patient, which may lead us to underestimate the incidence of AKI; Second, the definition of AKI in this study is limited to serum creatinine since it was impossible to evaluate AKI through continuous observation of urine output due to the limited medical conditions at that time. Finally, the exclusion of patients without repeat evaluation of renal function may cause overestimation of the incidence of AKI.

In conclusion, we validated the UCSD-Mayo risk score performed well in predicting hospital-acquired AKI in COVID-19 patients based on our large Chinese patients. The relatively poor performance of UCSD-Mayo risk score in ICU patients or patients with mechanical ventilation suggested new risk factors should be studied in these patients.

## Ethics approval and consent to participate

The study was performed in accordance with the Helsinki Declaration of 1975, and was approved by the Institutional Review Board of the Ruijin Hospital, Shanghai Jiao Tong University School of Medicine [ETHICS No: (2020) Linlun no.34th]. Informed consent was waived by the Research Ethics Commission.

## Data Availability

The datasets used and/or analyzed during the current study are available from the corresponding author on reasonable request.
